# Quantification of Cardiovascular Disease Risk Among Hypertensive Subjects in Active Romanian Population Using New Echocardiographic, Biological and Atherogenic Markers

**DOI:** 10.3390/medicina62010032

**Published:** 2025-12-24

**Authors:** Calin Daniel Popa, Rodica Dan, Iosef Haidar, Cristina Popescu, Roxana Dan, Tabita Popa, Lucian Petrescu

**Affiliations:** 1 Saint Luca of Crimea Medical Centre, 041915 București, Romaniaroxana.dan@gmail.com (R.D.);; 2 Department VI—Cardiology, General Medicine Faculty, University of Medicine and Pharmacy “Victor Babes” Timisoara, 300041 Timisoara, Romania; 3 Department of Life Sciences and Biology, General Medicine Faculty, “Vasile Goldis” Western University of Arad, 310328 Arad, Romania

**Keywords:** hypertension, atherogenic risk, cardiovascular risk, PON1, homocysteine

## Abstract

*Background and Objectives:* The objective of this study is to assess the efficacy of a novel software risk score, PulsIn, in predicting cardiovascular diseases within an independent study conducted on subjects from the western region of Romania. Accurate prediction of cardiovascular events in hypertensive patients remains challenging when relying solely on traditional risk scores. This study proposes PulsIn, a composite risk score that integrates classical, echocardiographic, inflammatory, renal, and metabolic markers, combined with machine learning, to refine cardiovascular risk stratification. *Materials and Methods:* In a prospective cohort of 300 hypertensive adults without prior major cardiovascular events, we collected demographic and clinical data, standard risk factors, laboratory biomarkers (including homocysteine, paraoxonase-1 activity, microalbuminuria, and lipid profile), and advanced echocardiographic parameters (3D left ventricular ejection fraction, diastolic function, global longitudinal strain, and left atrial strain). PulsIn was constructed as an extended composite score and used as input to machine learning models (random forest, XGBoost, and other tree-based algorithms) to predict incident major cardiovascular events. Model performance was assessed by receiver operating characteristic curves, discrimination, calibration, and feature importance and compared with established risk scores (SCORE2, Framingham, QRISK, and others). *Results:* PulsIn-based models showed improved predictive performance compared with traditional scores, with XGBoost and random forest achieving area under the curve values up to approximately 0.85–0.88, versus 0.60–0.78 for conventional scores. Echocardiographic indices of subclinical cardiac damage, microalbuminuria, homocysteine, and paraoxonase-1 activity emerged as key predictors, particularly enhancing reclassification in patients at intermediate risk by traditional tools. *Conclusions:* The PulsIn composite risk score, integrating multimodal clinical, echocardiographic, and biomarker data within a machine learning framework, offers more accurate cardiovascular risk prediction than conventional algorithms in hypertensive patients. External validation in larger, independent, and more diverse populations is required before routine clinical implementation.

## 1. Introduction

Currently, cardiovascular diseases remain the leading cause of mortality worldwide. Unfortunately, Romania ranks high on this scale, with a mortality rate of 62%, according to the European Registry of Cardiovascular Diseases and SEPHAR studies (III and IV) [1,2,3]. Identifying risk factors to develop and apply a therapeutic and effective strategy is one of the main objectives of researchers in the field, aiming for a more proper follow-up of each patient.

Identifying predictive markers that can be translated into preventive measures would substantially contribute to reducing the number of patients newly diagnosed with cardiovascular diseases (which are currently on an upward trend). Additionally, doctors require and will continue to require an accurate and reliable tool to assist them in identifying patients at high risk of experiencing a major cardiovascular event.

Several risk scores have been developed to estimate a patient’s 10-year risk of cardiovascular disease based on a certain number of risk factors. Examples include the Framingham risk score and the Reynolds risk score, both developed using patient data from the US.

Another well-known example is the SCORE risk system (SCORE 2 and SCORE 2-OP), which utilizes only a few classical risk factors (age, sex, smoking, SBP, and non-HDL cholesterol in SCORE 2) to estimate the 10-year fatal and non-fatal cardiovascular disease risk. It is applied differently across various countries, depending on the risk region in European countries [4,5,6,7,8,9].

The primary objective of our study was to show that discovering an improved estimation tool (incorporating more patient data related to cardiovascular risk) for predicting future major cardiovascular events in individuals with risk factors could significantly enhance risk assessment. This, in turn, would enable us to identify eligible individuals/patients who require a more proactive approach.

## 2. Materials and Methods

### 2.1. Study Design

This was an observational, prospective study conducted between 2017 and 2022 in a cohort of hypertensive patients with varying cardiovascular risk profiles. The study was designed to evaluate the predictive value of an extended panel of clinical, echocardiographic, and biochemical parameters for major cardiovascular events and to develop a composite cardiovascular risk score (PulsIn) adapted to the regional patient population.

The study protocol was conducted in accordance with the principles of the Declaration of Helsinki. All participants provided written informed consent prior to inclusion.

### 2.2. Study Population

A total of 300 subjects were consecutively enrolled and allocated into four groups according to their cardiovascular risk status:

1. Control group (C): Newly diagnosed hypertensive patients without known major cardiovascular events (n = 60).
2. Group P1: Hypertensive patients with concomitant dyslipidemia and type 2 diabetes mellitus (n = 80).
3. Group P2: Hypertensive patients with type 2 diabetes and dyslipidemia, with a documented history of at least one major cardiovascular event (coronary heart disease, stroke, or peripheral artery disease) and/or atrial fibrillation/flutter (n = 80).
4. Group P3: Hypertensive patients, with or without type 2 diabetes, who had recently experienced a major cardiovascular event (coronary heart disease, stroke, or peripheral artery disease) and/or atrial fibrillation/flutter (n = 80).

Inclusion criteria were:

- age between 35 and 85 years;
- diagnosis of arterial hypertension according to current guidelines;
- ability to provide informed consent.

Exclusion criteria were:

- type 1 diabetes mellitus;
- history of any malignancy prior to enrolment;
- severe non-cardiovascular comorbidities limiting life expectancy or precluding follow-up;
- poor echocardiographic window precluding reliable strain analysis.

Demographic and baseline clinical characteristics were recorded for all participants, including age, sex, body mass index (BMI), smoking status, blood pressure, and relevant comorbidities.

### 2.3. Data Collection and Clinical Assessment

All participants underwent a standardized clinical evaluation at inclusion. Medical history was obtained through structured anamnesis, focusing on cardiovascular risk factors and prior cardiovascular events. A physical examination was performed, including measurement of weight, height, and blood pressure.

Blood pressure was measured in accordance with the Guidelines of the European Society of Hypertension [10]. After at least 5 min of rest in the seated position, three consecutive measurements were obtained using a validated automatic sphygmomanometer, and the mean value was used for analysis. In selected cases, 24 h ambulatory blood pressure monitoring (ABPM) was performed to obtain a more comprehensive assessment of blood pressure profile and hypertension control.

An electrocardiogram (ECG) was recorded in all patients to identify rhythm abnormalities (atrial fibrillation/flutter), signs of left ventricular hypertrophy, or prior myocardial infarction when evident.

Information on current medication (antihypertensive, antidiabetic, lipid-lowering, antiplatelet or anticoagulant therapy) was collected from patient records and direct interviews.

### 2.4. Laboratory and Biomarker Assessment

Blood samples were drawn after an overnight fast. Standard biochemical analyses were performed in the local laboratory using routine automated methods. The following parameters were determined:

- Lipid profile: total cholesterol (TC), triglycerides (TG), low-density lipoprotein cholesterol (LDL-C), high-density lipoprotein cholesterol (HDL-C), non-HDL cholesterol, and total cholesterol/HDL-C ratio.
- Glycemic and metabolic markers: fasting plasma glucose, glycated hemoglobin (HbA1c), and serum homocysteine.
- Renal function and albuminuria: serum creatinine, uric acid, urinary albumin/creatinine ratio, presence of microalbuminuria, and estimated glomerular filtration rate (eGFR), calculated using a standard formula [11,12,13].
- Inflammatory marker: high-sensitivity C-reactive protein (CRP).
- Atherogenic enzyme marker: paraoxonase 1 (PON1) activity.

All laboratory measurements were performed according to manufacturer instructions and internal laboratory quality control procedures.

### 2.5. Echocardiographic Assessment

Transthoracic echocardiography was performed in all participants using PHILIPS EPIQ CVx system, following current recommendations [14,15,16,17]. 3D LVEF was evaluated using Philips’s automated dynamic quantification software (Philips CVx version 10, Philips Dynamic Heart Model (HM)) [18]. LV systolic dysfunction was defined as EF below 50%. GLS was measured from the apical two-, three-, and four-chamber views, and LA strain was measured from the apical four-chamber view using a semi-automated assessment tool with AutoStrain (TomTec Imaging Systems), with patients in the left lateral decubitus position. Two-dimensional, Doppler, and speckle-tracking analyses were carried out by experienced cardiologists who were blinded to clinical and laboratory data.

Conventional echocardiographic parameters included:

- left ventricular ejection fraction (LVEF, 2D);
- left ventricular dimensions and wall thickness;
- left atrial size;
- indices of diastolic function (E/A ratio, E/e’, deceleration time, left atrial size, and additional parameters as required).

Diastolic function was classified according to current guideline recommendations.

Advanced echocardiographic analysis included:

- Global longitudinal strain (GLS): Assessed by 2D speckle-tracking echocardiography using apical views (four-chamber, two-chamber, and long-axis). The endocardial border was manually traced, and tracking was automatically performed, with manual adjustments as necessary. A GLS value less negative than −19% (i.e., >−19%) was considered abnormal based on vendor-specific reference ranges.
- Left atrial (LA) strain: LA reservoir strain was measured using speckle-tracking from apical four-chamber views focused on the left atrium. A value below 35% was considered abnormal, based on the lower limit of the 95% confidence interval observed in the control group and supported by literature data.
- Three-dimensional LVEF (3D LVEF): When available, a 3D Heart Model was used to assess left ventricular volumes and ejection fraction, offering a more accurate quantification of systolic function [19,20].

All echocardiographic images were stored digitally and analyzed offline. For strain measurements, only images with adequate tracking quality in the majority of segments were accepted.

### 2.6. Definition of Outcomes

Major cardiovascular events were defined as the composite of:

- coronary heart disease (including myocardial infarction and documented coronary artery disease requiring revascularization or associated with significant stenosis);
- ischemic or hemorrhagic stroke;
- peripheral artery disease requiring intervention or documented by imaging; and/or
- the presence of atrial fibrillation or atrial flutter.

For the purposes of model development, patients with a documented prior major cardiovascular event or recent event (as defined above) were categorized as having an event, while those without such history were categorized as event-free.

### 2.7. Risk Score Development (PulsIn)

To develop an extended cardiovascular risk model, we used the open-source QRISK2-2014 equation as a foundational framework. QRISK2 integrates multiple clinical variables to estimate the 5- or 10-year risk of major cardiovascular events. We adapted this approach to our population and augmented it with additional parameters considered clinically relevant and available in our dataset.

The following categories of variables were considered for inclusion in the PulsIn model:

- classical clinical risk factors (age, sex, smoking status, blood pressure, diabetes, lipid profile);
- echocardiographic parameters (LVEF, diastolic dysfunction, GLS, LA strain, 3D LVEF);
- inflammatory marker (CRP);
- atherogenic markers (non-HDL cholesterol, total cholesterol/HDL-C ratio, PON1);
- renal function markers (creatinine, uric acid, albumin/creatinine ratio, microalbuminuria, eGFR) [21,22,23,24];
- metabolic marker (homocysteine) [25,26];
- presence of arrhythmias (atrial fibrillation/flutter).

These variables were used as candidate predictors in the statistical and machine learning models described below.

### 2.8. Statistical Analysis

All statistical analyses were performed using GraphPad Prism 9 and Python-based tools (version 3.11) for machine learning [27].

Continuous variables are presented as mean ± standard deviation (SD), while categorical variables are summarized as counts and percentages. Group comparisons were initially performed using:

- Student’s t-test or one-way ANOVA for normally distributed continuous variables;
- Mann–Whitney U test or Kruskal–Wallis test for non-normally distributed variables;
- χ^2^ test or Fisher’s exact test for categorical variables, as appropriate.

A *p*-value < 0.05 was considered statistically significant. Results with *p* < 0.01 were considered highly significant, and those with *p* < 0.001 were very highly significant.

For risk modeling, we used:

- Logistic regression to estimate the association between predictors and the presence of major cardiovascular events and to obtain odds ratios with 95% confidence intervals.
- Random forest and XGBoost classifiers as ensemble machine learning methods to capture non-linear relationships and interactions between variables.

Model performance was assessed by:

- area under the receiver operating characteristic curve (AUC-ROC);
- sensitivity, specificity, and accuracy at selected probability thresholds;
- calibration plots where applicable.

Feature importance was examined for the random forest and XGBoost models to identify the most influential predictors in the extended PulsIn score, with special attention to echocardiographic (diastolic dysfunction, GLS, LA strain) and biomarker parameters (CRP, PON1, homocysteine, renal markers).

All analyses were conducted on the complete dataset after appropriate data cleaning and encoding. Missing data were handled according to predefined rules (e.g., exclusion of cases with critical missing variables or imputation where justified), ensuring consistency across models.

## 3. Results

### 3.1. Baseline Clinical Characteristics

Baseline characteristics of the study population are summarized in Table 1. The mean age of male patients was 55.4 ± 2.5 years, whereas female patients had a mean age of 60.8 ± 1.7 years. The youngest subgroup was the control group, with a mean age of 45.7 ± 8.8 years in men and 48.4 ± 5.7 years in women. Overall, 21% of participants were older than 70 years, and among these, 66% were women. Approximately 75% of the cohort had a body mass index (BMI) greater than 25 kg/m^2^, and more than half were obese (BMI > 30 kg/m^2^), with 54% of the obese subjects being men. Regarding hypertension (HT), the mean systolic blood pressure (SBP) and diastolic blood pressure (DBP) values were 147/91 mmHg and were higher in male subjects compared to females (147.5/89.9 vs. 145/86.4 mmHg), and they increased with age in both male and female subjects. Group P3 exhibited better-controlled blood pressure values, possibly due to recent cardiovascular events, leading to more closely monitored treatment for hypertension and other comorbidities (Table 1).

### 3.2. Laboratory and Echocardiographic Parameters

We observed a more adverse lipid profile in subjects from the P2 group, characterized by higher total cholesterol, LDL cholesterol, triglycerides, and non-HDL cholesterol compared with the control group (C) and P1 (Table 2). In parallel, male patients in the P2 group showed higher levels of microalbuminuria, which likely reflect early glomerular damage in the setting of hypertension and suboptimal glycemic control. Notably, the differences in total cholesterol (TC), triglycerides (TG), and HDL cholesterol across the study groups were more clearly captured by non-HDL cholesterol, particularly among male patients, underscoring its value as an integrative marker of atherogenic lipoprotein burden (see Table 2).

Compared with the control group, P2 and P3 had significantly higher TC, LDL cholesterol, TG, and non-HDL cholesterol (all *p* < 0.05), with a stepwise increase across C, P1, P2, and P3.

The incidence of diabetes mellitus was 40%, with a higher prevalence in men (55%), considering that group P1 exclusively consisted of diabetic subjects. These patterns are consistent with progressive cardiometabolic risk across the study groups and may partly explain the higher incidence of major cardiovascular events observed in P3 (Table 2).

For the estimation of glomerular filtration rate (GFR), we used a free online calculator implementing the Modification of Diet in Renal Disease (MDRD) equation, as recommended by the International Society of Nephrology (ISN) [20]. Based on anamnesic data and serum creatinine levels, we calculated the estimated GFR (mL/min/1.73 m^2^) and classified kidney function for all study groups according to established chronic kidney disease (CKD) staging guidelines [21,23].

Approximately 60% of subjects in the P1 and P2 groups were classified as being in CKD stage I (with onset of microalbuminuria), whereas more than 55% of patients in the P3 group were in CKD stage II, with albumin/creatinine ratios consistent with clinical proteinuria, indicating the presence of renal dysfunction (see Table 2).

Another important parameter studied was hyperhomocysteinemia, as it is recognized as an independent risk factor for both women (even before menopause) and men, contributing to an increased risk of CV, venous thrombosis, and pregnancy complications. Additionally, a 5 μmol/L increase in homocysteine levels is associated with an elevated risk of coronary heart disease [24,25]. The mean values for homocysteine were 12.53 ± 6.52 μmol/L for female subjects and 14.13 ± 6.26 μmol/L for male subjects. Significant differences were observed at values above the reference range in study groups P2 and P3. Specifically, men exhibited significantly higher levels of homocysteine compared to women, with values exceeding the normal range.

Regarding results from the human paraoxonase 1 (PON1), the serum levels were lower in the male group compared to the female group. Similarly, smokers (13.3% male and 7.6% female) exhibited lower PON1 activity levels compared to non-smokers. Both males and females in groups P1 and P3 had low levels of PON1 activity (P1 female vs. P3 female: 386.6 ± 16.1 vs. 344.7 ± 6.9, *p* < 0.008).

**Figure 1:**
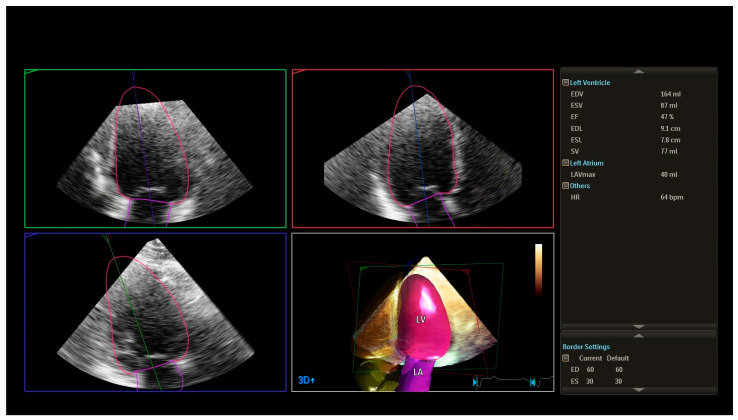
HM (heart model) with advanced 3D speckle technology which follows the heart’s movement throughout each frame of the cardiac cycle. By analysing dynamic contours of the left atrium (LA) and left ventricle (LV), along with waveforms, LV and LA indexes, and LV mass measurements, the system offers a comprehensive view of left heart function. (Top right): are End-diastolic volume (EDV) = 164 mL, End-systolic volume (ESV) = 87 mL, Ejection fraction (EF) = 47%, stroke volume (SV) = 77 mL, end-diastolic length (EDL) = 9.1 cm, end-systolic length (ESL) = 7.8 cm. Three-dimensional rendering of the left ventricle (pink) and left atrium (purple); maximum left atrial volume (LAV_max_) = 40 mL. Heart rate = 64 bpm. The mildly reduced ejection fraction and moderately dilated left ventricle, combined with severely reduced global longitudinal strain (Figure 2), illustrate subclinical myocardial impairment detectable by advanced echocardiographic imaging.

The left ventricular ejection fraction (LVEF, 3D Heart Model) was predominantly above 50% in the control group (C) and group P1, indicating largely preserved systolic function in these patients [see Figure 1]. In contrast, groups P2 and P3 exhibited mildly reduced LVEF values (approximately 45–50%), especially among older individuals with diabetes and inadequately controlled blood glucose levels. The lowest LVEF values were observed in the P3 group, which comprised patients who had experienced a major cardiovascular event, such as myocardial infarction, ischemic stroke, or coronary artery bypass grafting (CABG) (Table 3).

**Figure 2:**
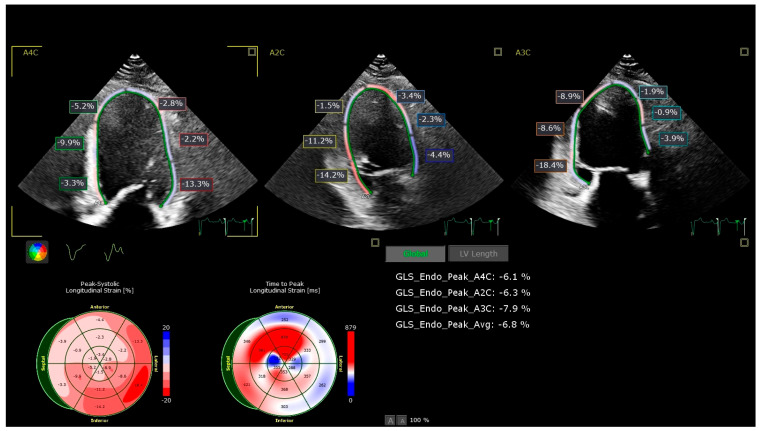
GLS from P3 study group. Speckle-tracking echocardiographic assessment of left ventricular longitudinal strain. Top row: Apical four-chamber (A4C), two-chamber (A2C), and three-chamber (A3C) views with color-coded segmental strain values and strain curves. Bottom left: Bull’s-eye plot of peak systolic longitudinal strain (%), showing regional heterogeneity with reduced strain (less negative values, red) in multiple segments. Bottom right: Bull’s-eye plot of time to peak longitudinal strain (ms), illustrating mechanical dyssynchrony. Right panel: Summary of global longitudinal strain (GLS) values; average endocardial GLS = −6.8%, indicating severely reduced myocardial function despite preserved left ventricular geometry.

GLS (global longitudinal strain) and LA strain are relatively new echocardiographic parameters that we aimed to incorporate into our cardiovascular disease (CVD) risk score calculator. The lowest values for both were recorded in 60% of the P2 group and over 80% of the P3 group. In the control group, GLS values were normal in both genders. However, for the P1 group, GLS values were slightly decreased, especially in patients with higher blood pressure values, irrespective of gender [see Figure 2].

**Figure 3:**
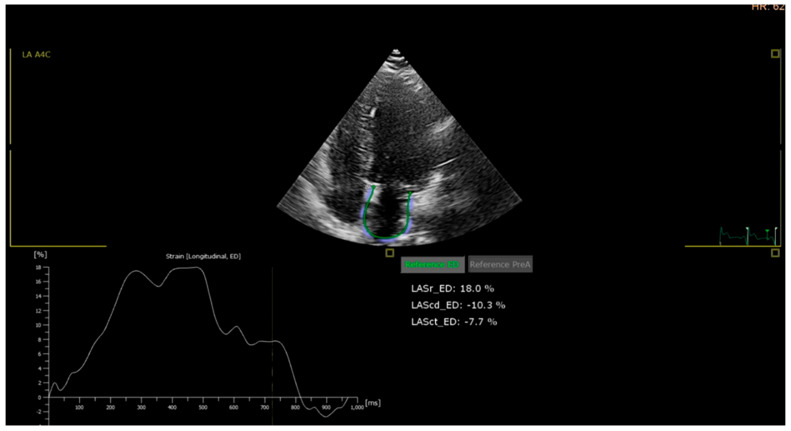
LA strain from a patient from P3 study group. Left atrial strain analysis by speckle-tracking echocardiography. (**Bottom Left**): Strain-time curve showing longitudinal deformation over the cardiac cycle. (**Center Right**): Summary of left atrial strain parameters; LA reservoir strain (LASr_ED) = 18.0%, LA conduit strain (LAScd_ED) = −10.3%, LA contractile strain (LASct_ED) = −7.7%. Reduced LA reservoir function indicates impaired atrial compliance and is associated with diastolic dysfunction, increased risk of atrial fibrillation, and adverse cardiovascular outcomes.

The LA strain parameter showed values within normal limits for the control group. However, for groups P1 (with an example in Image 3) and P2, there were slightly altered values, even though the LVEF remained unchanged. This suggests that atrial septum (AS) performance during all phases of LA activity—the tank, pipe, and pump—is already affected, indicating the early stages of subclinical heart failure [see Figure 3].

After establishing all parameters and collecting the necessary data, we proceeded to include them in our cardiovascular disease (CVD) risk calculator (PulsIn). This involved incorporating both biochemical parameters and echocardiographic parameters (such as GLS, LA strain, HM, and diastolic dysfunction), as well as biochemical and atherogenic markers (PON1). Subsequently, we calculated the risk status for each parameter using logistic regression analysis.

### 3.3. Model Performance and Feature Importance

#### 3.3.1. Logistic Regression Model

In multivariable logistic regression analysis, several variables emerged as independent predictors of major cardiovascular events. Traditional risk factors such as age, obesity, diabetes, and renal disease were all significantly associated with higher event risk. In addition, echocardiographic parameters (diastolic dysfunction, GLS, LA strain, and 3D ejection fraction by Heart Model) and biochemical markers (homocysteine and PON1) showed strong associations, with the largest odds ratios observed for LA strain, homocysteine, GLS, and HM. Gender and smoking did not remain significant predictors after adjustment, suggesting that the extended echocardiographic and biomarker profile provides incremental prognostic information beyond conventional risk factors.

Multivariable logistic regression was performed using a simultaneous (enter) method, with the occurrence of major cardiovascular events as the dependent variable and gender, age, smoking status, obesity, diabetes, renal disease, diastolic dysfunction (DD), global longitudinal strain (GLS), left atrial (LA) strain, HeartModel 3D ejection fraction (HM), homocysteine, and PON1 as covariates; odds ratios (Exp(B)) with 95% confidence intervals were calculated, and a two-sided *p*-value < 0.05 was considered statistically significant (Table 4).

#### 3.3.2. Random Forest Model

The random forest model showed better discriminative performance than logistic regression, with an AUC-ROC of 0.86 (95% CI: 0.81–0.90), sensitivity of 80%, specificity of 78%, and accuracy of 79% (Table 4). This represented a statistically significant improvement over the reference model (*p* = 0.01), indicating that the random forest algorithm more accurately identified patients at risk of major cardiovascular events.

**Figure 4:**
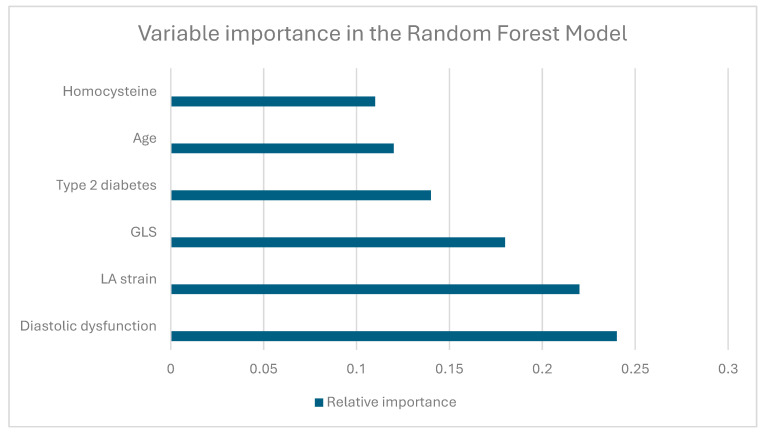
Variable importance ranking in the random forest model.

Relative importance of the top predictors in the random forest model: diastolic dysfunction, left atrial (LA) strain, global longitudinal strain (GLS), presence of type 2 diabetes, age, and homocysteine levels were among the most influential variables for predicting major cardiovascular events (Figure 4).

#### 3.3.3. XGBoost Model

The XGBoost model demonstrated the best overall discriminative performance among the evaluated algorithms, with an AUC-ROC of 0.88 (95% CI: 0.83–0.92), sensitivity of 82%, specificity of 80%, and accuracy of 81% (Table 5).

**Figure 5:**
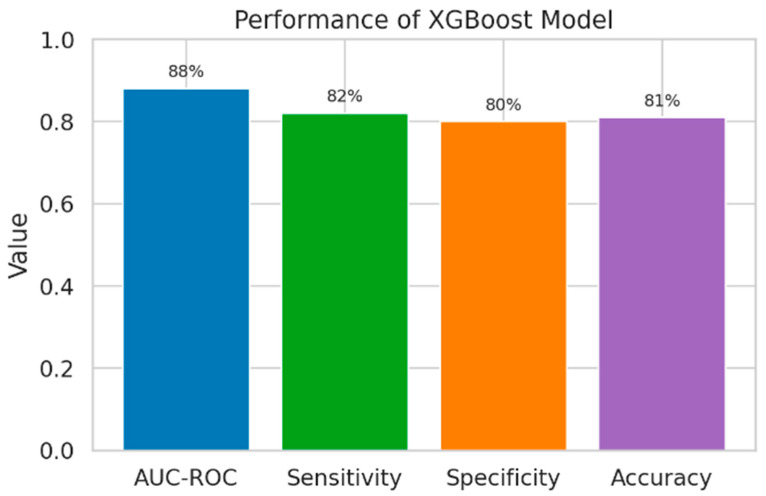
XGBoost model.

Compared with logistic regression (reference model), XGBoost provided a statistically significant improvement in model performance (*p* = 0.005), indicating a superior ability to correctly identify patients at risk of major cardiovascular events (Figure 5).

#### 3.3.4. Comparison of Model Performance

Receiver operating characteristic (ROC) curves of logistic regression, random forest, and XGBoost models for prediction of major cardiovascular events in the study cohort.

The PulsIn-based XGBoost model achieved the highest discriminative performance for prediction of major cardiovascular events (AUC = 0.88), followed by the random forest model (AUC = 0.86) and logistic regression (AUC = 0.78) (Table 5).

#### 3.3.5. CV Risk Score Comparison

In the overall study population, all four risk scores were significantly higher in patients who experienced cardiovascular events than in those who remained event-free. Mean values for PulsIn, Framingham, Qrisk2 and Score2 and Score2 OP were consistently and substantially increased in the event group, with Welch’s t-tests confirming statistically significant between-group differences for each score (all *p* values < 0.01) (Table 6).

When discrimination for cardiovascular events was evaluated using ROC analysis, all scores demonstrated at least moderate predictive ability, with areas under the curve (AUCs) exceeding 0.60. Among them, PulsIn showed the highest AUC, indicating the best overall discrimination in this dataset, closely followed by Score2 and Score2 OP.

Qrisk2 and Framingham yielded slightly lower but still clinically relevant AUCs. Thus, while all four scores were statistically associated with cardiovascular events, PusIn provided the strong separation between patients with and without events in our cohort (Table 6).

During the follow-up period (2017–2022), the overall incidence of cardiovascular disease (CVD) in the study cohort was 58%. Among patients in groups P2 and P3, the distribution of events was as follows: coronary heart disease 43.33%, stroke 4.33%, peripheral arterial disease (PAD) 4.0%, atrial fibrillation (AF) 7.33%, and all-cause mortality 2.33%. The incidence of these events increased with advancing age and was more prevalent among older participants, with rates exceeding 25% in the oldest age categories in both women and men.

## 4. Discussions

Utilizing established cardiovascular risk calculation models documented in the literature and supplemented with the data gathered in our office, we tried to construct a mathematical model that effectively integrates the features of known models, particularly those applicable to parameters obtainable in a clinical setting. Many models found in the literature exhibit a general structure [28,29,30,31] basically utilizing this formula:

$$P_{man}=100\left(1-{coef}^{expexp\;\left(L_{man}\right)\;}\right),$$

the $exp\;exp\;\left(a\right)\;=e^{a}$, e≈2.71 being the basis of natural logarithms, and coef is a coefficient specific to each formula and each gender, being, as a rule, a number between 0.95 and 0.99.

$$L_{man}=\sum_{Parameters}\beta_{Parameter}\;ln\;ln\;(Parameter),$$

The parameter represents the variables utilized, while β_Parameter_ denotes coefficients derived from studies in the literature, particularly for the Framingham and QRISK methods, which are widely recognized. These coefficients are interpolated from the studies for the model described above. The natural logarithm (*ln*), which represents the logarithm with base e (approximately 2.71), is also employed. While the formulas provided are applicable for male patients, for female patients, the formula has the same structure but with adapted coefficients: *coef*♀ and *β*♀, which reflect the pathophysiological differences between sexes.

The graphical user interface of the desktop application has been developed, and the model has been integrated into the PulsIn application. Subsequently, the application was employed to calculate the cardiovascular risk for the 300 patients in the office, yielding a risk interval for each patient.

### 4.1. Principal Findings

In this study, we showed that patients with established or impending cardiovascular disease (P2 and P3) exhibited a progressively more adverse profile of classical risk markers compared with controls and lower-risk groups (C and P1). Blood pressure, lipid parameters (particularly non-HDL cholesterol and triglycerides), markers of renal impairment (microalbuminuria, albumin/creatinine ratio, and lower estimated GFR), and glycemic control were all less favorable in the higher-risk groups. These differences were consistent with the graded increase in cardiovascular event rates observed during follow-up, supporting the internal coherence of the risk stratification.

Beyond traditional risk factors, we found that extended biomarkers and advanced echocardiographic parameters provided additional discriminatory value. Individuals in P2 and P3 not only had worse lipid and renal profiles but also showed more frequent diastolic dysfunction, impaired global longitudinal strain (GLS), reduced left atrial (LA) strain, and higher circulating homocysteine levels. These alterations were evident even when left ventricular ejection fraction was only mildly reduced or still within the lower normal range, indicating that subtle functional impairment and myocardial/atrial remodeling precede overt systolic dysfunction in this population.

Our PulsIn-based risk estimation performed favorably when compared with traditional clinical risk scores. While conventional tools derived from classical factors captured a substantial proportion of risk [32,33], the inclusion of PulsIn and extended markers improved risk discrimination and reclassification, particularly among patients in the intermediate-risk range. In other words, PulsIn helped to more accurately identify individuals who subsequently experienced major cardiovascular events, beyond what could be achieved by standard risk scores alone.

Taken together, these findings highlight that combining classical risk factors with extended biomarkers and detailed cardiac functional assessment—especially diastolic parameters, GLS, LA strain, and homocysteine—yields a more nuanced and clinically meaningful stratification of cardiovascular risk. This integrated approach better reflects the continuum from subclinical organ damage to overt cardiovascular events and may support earlier and more targeted preventive interventions.

### 4.2. Differences in Classical and Extended Markers Between Groups

In our cohort, we observed a clear, stepwise worsening of both classical and extended cardiovascular risk markers from the control group (C), through P1, to the higher-risk groups P2 and P3.

Patients in groups P2 and P3 had a more adverse profile of traditional risk factors compared with C and P1. Total cholesterol, LDL cholesterol, and especially non-HDL cholesterol and triglycerides were higher in the P1–P3 groups than in controls, with the most unfavorable values in P2 and P3. HDL cholesterol showed an opposite trend, with lower levels in the higher-risk groups. These differences were particularly evident in male subjects and were consistently reflected in non-HDL cholesterol, which better captured the overall atherogenic burden than LDL cholesterol alone.

Markers of renal involvement also differed between groups. Microalbuminuria and the albumin/creatinine ratio were more frequently elevated in P2 and P3, while estimated GFR was lower, placing a significant proportion of these patients into early chronic kidney disease stages. Thus, P2 and P3 combined a clustering of dyslipidemia, impaired glucose control, hypertension, and early renal damage, in line with their higher incidence of cardiovascular events during follow-up.

Beyond these classical factors, extended markers and advanced echocardiographic parameters showed additional, clinically meaningful differences between the groups. Patients in P2 and P3 more often had diastolic dysfunction and subclinical myocardial impairment, as evidenced by reduced global longitudinal strain (GLS) and lower left atrial (LA) strain, even when left ventricular ejection fraction was preserved or only mildly reduced. These findings indicate that subtle myocardial and atrial remodeling is already present in higher-risk patients before overt systolic dysfunction becomes apparent.

Moreover, extended biochemical markers, such as homocysteine, were higher in the P2 and P3 groups and paralleled the burden of other risk factors and organ damage. Together with microalbuminuria and alterations in GLS and LA strain, these extended markers helped to identify individuals at greater cardiovascular risk beyond what could be inferred from classical markers alone.

In summary, the progression from C to P1, P2, and P3 was associated with a gradual deterioration in traditional risk factors and a parallel worsening in extended biomarkers and functional cardiac parameters, supporting an integrated, multimodal approach to cardiovascular risk stratification in this population.

### 4.3. Performance of PulsIn vs. Traditional Risk Estimation

In our study, the PulsIn-based approach outperformed traditional cardiovascular risk estimation models that rely primarily on classical risk factors (age, sex, blood pressure, lipids, smoking, and diabetes status). While conventional scores captured a substantial proportion of high-risk individuals, they tended to underestimate risk in patients with early organ damage or subclinical cardiac dysfunction [34,35].

PulsIn, which integrates extended markers (such as microalbuminuria and homocysteine) and advanced echocardiographic parameters (including diastolic dysfunction, GLS and LA strain), showed better discrimination and reclassification of patients who later developed major cardiovascular events. Compared with traditional risk scores, PulsIn identified a larger share of individuals in P2 and P3 who actually experienced coronary events, stroke, PAD, atrial fibrillation, or death during follow-up.

In practical terms, this means that patients classified as “intermediate risk” by standard scores were more accurately stratified when PulsIn was applied: some were correctly reclassified to higher risk, in line with their subsequent event rates, while others were downgraded when extended markers did not indicate significant subclinical damage. Overall, PulsIn provided a more nuanced and clinically relevant risk profile than traditional estimation alone, supporting its potential value as an adjunct tool for individualized cardiovascular risk assessment [36].

### 4.4. Importance of the New Parameters That Were Included in PulsIn Calculator

In our cohort, subclinical cardiac dysfunction and extended biochemical markers added important prognostic information beyond classical risk factors.

From a functional standpoint, many patients in the higher-risk groups (P2 and especially P3) already showed diastolic dysfunction despite having preserved or only mildly reduced LVEF. This pattern suggests that impaired relaxation and increased ventricular stiffness appear early in the disease continuum, before overt systolic failure becomes evident. The presence of diastolic dysfunction in P2–P3 paralleled the accumulation of traditional risk factors and the higher incidence of cardiovascular events during follow-up.

Global longitudinal strain (GLS) and left atrial (LA) strain further refined this picture. Both parameters were consistently more impaired in P2 and P3 compared with C and P1, even when LVEF remained above conventional thresholds. Reduced GLS pointed to early longitudinal myocardial fiber involvement, while lower LA strain reflected increased left ventricular filling pressures and atrial remodeling. Together, these strain-based indices captured subtle myocardial and atrial damage that standard echocardiographic parameters would have underestimated.

On the biochemical side, homocysteine levels were higher in the advanced groups and tracked with the burden of other risk factors and markers of organ damage, including microalbuminuria and reduced GFR. Elevated homocysteine likely reflects a pro-atherogenic and pro-thrombotic background, providing a mechanistic link between metabolic disturbance, endothelial dysfunction, and the observed increase in coronary events, stroke, and peripheral arterial disease.

Overall, the combination of diastolic dysfunction, impaired GLS and LA strain, and elevated homocysteine identified patients with an unfavorable subclinical profile, even when traditional markers alone might have suggested only moderate risk. These extended functional and biochemical markers therefore helped explain why patients in P2 and P3 experienced a higher rate of major cardiovascular events, underscoring their potential value for earlier and more accurate cardiovascular risk stratification.

In addition to homocysteine and markers of renal impairment, we also evaluated paraoxonase-1 (PON1), an HDL-associated enzyme with antioxidant and anti-atherogenic properties. PON1 activity was lower in patients from the P2 and P3 groups compared with controls and P1, paralleling the more adverse lipid profile and higher burden of cardiovascular events in these groups. This reduction in PON1 suggests impaired HDL functionality rather than a simple quantitative HDL cholesterol deficit and may partly explain the limited protective effect of HDL observed in high-risk patients. When considered alongside homocysteine, microalbuminuria, and strain-based echocardiographic parameters, decreased PON1 activity further supports the presence of heightened oxidative stress and endothelial dysfunction in individuals at greatest cardiovascular risk. 

### 4.5. Comparison with Previous Studies

Our findings are broadly consistent with large-scale cardiovascular risk algorithms such as Framingham, SCORE2, and QRISK, which all emphasize age, blood pressure, lipids, smoking, and diabetes as major drivers of risk. In line with these models, our higher-risk groups (P2 and especially P3) showed clustering of classical risk factors: adverse lipid profile (higher non-HDL cholesterol and triglycerides, lower HDL), hypertension, and poor glycemic control, and these were accompanied by higher rates of incident CVD during follow-up.

However, results also highlight important limitations of traditional tools. Framingham, Score2 and Score2 OP, and Qrisk-type scores primarily quantify clinical risk based on baseline factors, but they do not routinely account for subclinical organ damage or extended biomarkers [37,38,39,40,41]. In our cohort, parameters such as microalbuminuria, reduced eGFR, PON1 activity, homocysteine, and strain-based echocardiographic indices (GLS, LA strain) captured additional risk that was not fully explained by classical variables alone. This is in line with recent reports showing that standard risk scores tend to underestimate risk in patients with early kidney disease, subtle myocardial dysfunction, or metabolic/inflammatory activation, and supports the added value of integrating organ damage markers into risk models [42,43,44].

Our PulsIn-based approach, which incorporates these extended measures, therefore complements rather than replaces Framingham/SCORE2/QRISK: it refines risk stratification, particularly in the “intermediate risk” range, where decision-making about intensifying therapy is most challenging [45].

Observations that diastolic dysfunction, reduced GLS, and impaired LA strain are more frequent and more severe in P2 and P3 are in good agreement with prior echocardiographic studies. Kalam et al. have shown that global longitudinal strain predicts major adverse cardiovascular events and heart failure hospitalizations even when left ventricular ejection fraction is preserved [46]. Similarly, impaired LA strain has been associated with incident atrial fibrillation, stroke, and heart failure, as it reflects chronically elevated filling pressures and atrial remodeling.

Our data reinforce these findings in a mixed cardiometabolic population: patients with worse GLS and LA strain had higher event rates and clustered in the groups with more advanced risk profiles and established CVD (P2–P3). Importantly, this occurred even in subjects with only mildly reduced or near-normal LVEF, aligning with prior literature that strain-based parameters detect subclinical myocardial and atrial dysfunction earlier than conventional measures. Thus, our results support the growing body of evidence that GLS and LA strain should be considered as prognostic markers in routine risk assessment, particularly in patients with diabetes, hypertension, or multiple risk factors.

The findings regarding PON1, homocysteine, and microalbuminuria fit well within existing evidence linking oxidative stress, endothelial dysfunction, and early renal damage to cardiovascular risk. Previous studies have demonstrated that low PON1 activity is associated with increased atherosclerotic burden and incident coronary events, reflecting impaired antioxidant function of HDL and reduced protection against LDL oxidation. In our work, lower PON1 activity in P2 and P3 mirrors this pattern and coincides with a more atherogenic lipid profile and a higher incidence of clinical events. This supports the concept that not only the quantity but also the functional quality of HDL is crucial for cardiovascular protection [47,48,49,50,51].

Similarly, elevated homocysteine has long been recognized as a marker of endothelial dysfunction and a pro-thrombotic, pro-atherogenic background. Our observation of higher homocysteine levels in the more advanced groups is concordant with prior meta-analyses and cohort studies, which reported associations between hyperhomocysteinemia and coronary artery disease, stroke, and peripheral arterial disease [52,53,54]. Although homocysteine-lowering therapies have shown mixed effects on hard outcomes, homocysteine elevation remains a robust marker of heightened vascular risk and metabolic stress.

Finally, results on microalbuminuria and the albumin/creatinine ratio are very much in line with large epidemiological studies (including in diabetes and hypertension), which consistently show that even mild increases in albumin excretion predict CVD events, heart failure, and mortality [55]. The higher prevalence of microalbuminuria and early CKD stages in P2 and P3 confirms that renal microvascular damage is an early and powerful integrator of cardiovascular risk, beyond classical factors.

Taken together, our study supports and extends the existing literature by showing that decreased PON1 activity, elevated homocysteine, and the presence of microalbuminuria cluster with impaired GLS/LA strain and classical risk factors in patients who ultimately experience more events. This integrated picture reinforces the idea that an approach focused solely on traditional risk factors underestimates the true burden of vascular and myocardial injury and that combining functional, biochemical, and structural markers—as we did with PulsIn—may offer a more accurate and clinically actionable assessment of cardiovascular risk.

### 4.6. Clinical Implications

Our findings suggest that PulsIn, or similar extended risk models, can substantially refine cardiovascular risk stratification beyond traditional tools such as Framingham, SCORE2, or QRISK. By integrating classical risk factors with organ damage markers (microalbuminuria, reduced eGFR), functional cardiac indices (diastolic dysfunction, GLS, LA strain), and biochemical markers of oxidative stress and endothelial dysfunction (homocysteine, PON1), PulsIn identifies patients whose “true” risk is higher than what standard scores alone would indicate.

This is particularly relevant in patients classified as intermediate risk by conventional models, where therapeutic decisions (e.g., initiation of high-intensity statins, SGLT2 inhibitors, GLP-1 receptor agonists, or more aggressive blood pressure targets) are often uncertain. In this group, the detection of impaired strain parameters, microalbuminuria, elevated homocysteine, or low PON1 activity can justifiably reclassify patients into a higher risk category, aligning their risk estimate with the observed incidence of major CVD events in our cohort.

Use of PulsIn-like extended models has direct implications for clinical follow-up. Patients with evidence of subclinical dysfunction—such as diastolic dysfunction, abnormal GLS or LA strain, early renal impairment, and adverse biomarker profiles—could be scheduled for closer and more structured surveillance, even if their traditional risk score is only moderate.

In practice, this might translate into shorter follow-up intervals for cardiology or internal medicine review, earlier and more frequent assessment of blood pressure, lipids, glucose control, and renal function, and targeted follow-up echocardiography (including strain analysis) in those with initial abnormalities.

Preventive strategies can also be more personalized. For example, the presence of microalbuminuria and reduced eGFR may support earlier use of RAAS blockade or SGLT2 inhibitors; impaired GLS/LA strain can prompt tighter blood pressure control and more aggressive anti-ischemic strategies; and elevated homocysteine or low PON1 may justify intensified lifestyle interventions and optimization of lipid-lowering therapy.

Finally, PulsIn helps to identify a subset of patients who may benefit from aggressive, multifactorial management despite not necessarily fulfilling the highest risk categories in standard algorithms. In our study, patients in P2 and especially P3 combined unfavorable classical risk factors, evidence of early renal and vascular injury (microalbuminuria, lower eGFR), subclinical myocardial and atrial dysfunction (diastolic dysfunction, reduced GLS, impaired LA strain), and biochemical evidence of increased oxidative and endothelial stress (elevated homocysteine, reduced PON1).

This clustering of abnormalities is precisely the profile that justifies earlier and more intensive intervention, including high-intensity statin therapy, strict blood pressure and glycemic targets, smoking cessation programs, weight reduction strategies, and potentially broader use of cardioprotective agents.

In summary, the clinical implication of our results is that a PulsIn-type extended model can move risk assessment from a purely statistical prediction based on demographic and clinical factors to a pathophysiology-oriented, organ damage-focused approach, enabling more accurate risk stratification, tailored follow-up intensity, and timely identification of patients who should receive aggressive preventive management.

### 4.7. Strengths and Limitations

A major strength of this study is the use of a well-characterized cohort, with systematic collection of clinical, biochemical, and echocardiographic data and prospective follow-up for hard cardiovascular outcomes. This comprehensive phenotyping allowed us to capture not only classical risk factors but also early organ damage and subclinical cardiac dysfunction.

Another important strength is the inclusion of advanced echocardiographic techniques, particularly global longitudinal strain (GLS) and left atrial strain, together with standardized assessment of diastolic function. These parameters are rarely integrated into routine risk scores, yet in our cohort, they provided additional, clinically meaningful information beyond left ventricular ejection fraction.

The study also benefits from the combination of multiple marker domains—classical risk factors, renal and vascular damage (microalbuminuria, eGFR), extended biochemical markers (homocysteine, PON1), and functional cardiac indices—within a single framework. This multi-layered approach reflects the complex pathophysiology of cardiovascular disease more faithfully than models restricted to demographic and basic laboratory variables.

Finally, we evaluated multiple predictive models, including traditional risk tools and the PulsIn-based extended model, and directly compared their performance. This allowed us to position PulsIn not as a theoretical construct but as a practical alternative or complement to existing risk scores, with documented gains in discrimination and reclassification.

Several limitations should be acknowledged. First, the sample size is modest compared with large population-based cohorts used to derive tools such as Framingham, Score2 and Score2 OP, or QRISK. This limits the precision of some estimates and may restrict the ability to fully explore subgroup effects or interaction terms.

Second, the study was conducted in a single region and healthcare setting, which may limit generalizability. Local patterns of risk factor distribution, treatment practices, and socioeconomic conditions could influence both baseline characteristics and event rates. As a result, the performance of PulsIn in other populations or healthcare systems remains to be confirmed.

Third, some components of the extended model, such as advanced echocardiography (GLS, LA strain), PON1 activity, and detailed biochemical profiling, may not be routinely available in all centers. Their use entails additional costs, technical expertise, and equipment, which could restrict widespread implementation, particularly in resource-limited settings.

Finally, although internal validation was performed, the PulsIn model requires external validation in independent cohorts before it can be recommended for routine clinical use. Calibration and performance may differ in populations with different baseline risk, ethnic composition, or treatment patterns. Future studies should also assess the clinical impact of using PulsIn in practice, including whether its use leads to changes in management and, ultimately, to better cardiovascular outcomes.

## 5. Conclusions

In this prospective cohort, an extended composite risk model (PulsIn) that integrates echocardiographic parameters (including diastolic dysfunction, GLS, and LA strain) with inflammatory, renal, and metabolic markers (such as microalbuminuria, eGFR, homocysteine, and PON1), together with classical risk factors, demonstrated superior predictive performance for incident cardiovascular events compared with traditional risk scores alone. Among the tested approaches, machine learning models—particularly random forest and XGBoost—provided the best discrimination and reclassification of patients into clinically relevant risk categories. These findings support the concept that a multidimensional, organ damage-oriented risk assessment better captures true cardiovascular risk; however, the PulsIn model now requires validation and calibration in larger, independent, and ethnically diverse cohorts before it can be broadly implemented in routine clinical practice.

## Figures and Tables

**Table 1:** Clinical and demographic characteristics of the study population.

Variable	Total (n = 300)	C (n = 60)	P1 (n = 80)	P2 (n = 80)	P3 (n = 80)	*p*-Value
Age, years	58.2 ± 9.4	52.1 ± 7.8	57.9 ± 8.6	60.3 ± 9.1	62.0 ± 9.8	<0.001
Male sex, n (%)	168 (56.0)	30 (50.0)	46 (57.5)	48 (60.0)	44 (55.0)	0.62
Systolic BP, mmHg	142 ± 16	138 ± 14	144 ± 15	145 ± 17	143 ± 16	0.04
Diastolic BP, mmHg	86 ± 9	84 ± 8	87 ± 9	88 ± 10	85 ± 9	0.08
Body mass index, kg/m^2^	29.4 ± 4.2	28.1 ± 3.9	30.2 ± 4.3	29.8 ± 4.1	29.6 ± 4.5	0.03
Current smoker, n (%)	96 (32.0)	18 (30.0)	25 (31.3)	26 (32.5)	27 (33.8)	0.94
Type 2 diabetes, n (%)	240 (80.0)	0 (0)	80 (100)	80 (100)	80 (100)	<0.001
Dyslipidemia, n (%)	240 (80.0)	0 (0)	80 (100)	80 (100)	80 (100)	<0.001
History of MACE, n (%)	160 (53.3)	0 (0)	0 (0)	80 (100)	80 (100)	<0.001
Atrial fibrillation/flutter, n (%)	90 (30.0)	0 (0)	10 (12.5)	40 (50.0)	40 (50.0)	<0.001

Legend: data are presented as mean ± standard deviation (SD) or number (percentage). BP, blood pressure; MACE—major adverse cardiovascular events.

**Table 2:** Biological data of study groups compared to the control group.

Variable	C (n = 60)	P1 (n = 80)	P2 (n = 80)	P3 (n = 80)	*p*-Value
Total cholesterol, mg/dL	210 ± 38	228 ± 42	230 ± 45	225 ± 40	0.02
LDL cholesterol, mg/dL	130 ± 32	142 ± 35	144 ± 36	140 ± 34	0.03
HDL cholesterol, mg/dL	49 ± 11	45 ± 10	44 ± 9	43 ± 9	0.01
Triglycerides, mg/dL	150 (110–190)	180 (140–230)	190 (150–240)	185 (145–235)	0.001
Non-HDL cholesterol, mg/dL	161 ± 37	183 ± 41	186 ± 43	182 ± 39	0.001
TC/HDL ratio	4.3 ± 1.1	5.1 ± 1.2	5.3 ± 1.3	5.2 ± 1.2	<0.001
CRP, mg/L	2.1 (1.0–3.4)	3.0 (1.8–4.8)	3.6 (2.1–5.9)	3.8 (2.3–6.2)	<0.001
Homocysteine, µmol/L	11.5 ± 3.0	13.2 ± 3.6	14.1 ± 3.8	14.4 ± 3.9	<0.001
PON1 activity, U/L	120 ± 35	105 ± 32	98 ± 30	95 ± 28	<0.001
Creatinine, mg/dL	0.9 ± 0.2	1.0 ± 0.2	1.1 ± 0.3	1.2 ± 0.3	<0.001
eGFR, mL/min/1.73 m^2^	92 ± 18	85 ± 16	80 ± 18	78 ± 19	<0.001
Albumin/creatinine ratio	12 (7–20)	20 (12–38)	30 (18–55)	32 (20–60)	<0.001
Microalbuminuria, n (%)	6 (10.0)	20 (25.0)	30 (37.5)	32 (40.0)	<0.001

Legend: data are presented as mean ± SD, median (interquartile range), or number (percentage), as appropriate. CRP—C-reactive protein; PON1—paraoxonase-1; eGFR—estimated glomerular filtration rate; TC, total cholesterol.

**Table 3:** Eco data of study groups compared to the control group.

Variable	C (n = 60)	P1 (n = 80)	P2 (n = 80)	P3 (n = 80)	*p*-Value
LV end-diastolic diameter, mm	49 ± 4	50 ± 5	51 ± 5	52 ± 5	0.01
LV mass index, g/m^2^	96 ± 18	104 ± 20	112 ± 22	115 ± 24	<0.001
LVEF (2D), %	60 ± 4	58 ± 5	55 ± 6	52 ± 7	<0.001
LVEF (3D), %	59 ± 5	57 ± 6	53 ± 7	50 ± 8	<0.001
Diastolic dysfunction, n (%)	10 (16.7)	30 (37.5)	50 (62.5)	55 (68.8)	<0.001
GLS, %	−20.5 ± 1.8	−19.0 ± 2.0	−17.5 ± 2.2	−16.8 ± 2.4	<0.001
Abnormal GLS (<−19%), n (%)	8 (13.3)	28 (35.0)	50 (62.5)	55 (68.8)	<0.001
LA volume index, mL/m^2^	26 ± 5	30 ± 6	34 ± 7	36 ± 8	<0.001
LA strain, %	42 ± 6	38 ± 7	34 ± 8	32 ± 8	<0.001
Abnormal LA strain (<35%), n (%)	6 (10.0)	24 (30.0)	48 (60.0)	52 (65.0)	<0.001

Legend: data are presented as mean ± SD or number (percentage). LV—left ventricle; LVEF—left ventricular ejection fraction; GLS—global longitudinal strain; LA—left atrium.

**Table 4:** Risk parameters for CVD.

	B	S.E.	Sig.	Exp(B)	95% C.I. for EXP(B)	
					Lower	Upper
Gender	0.104	0.302	0.531	1	0.499	1.629
Age	0.128	0.388	0.001	1.117	0.412	1.881
Obesity	0.115	0.328	0.006	1.191	0.469	1.695
Diabetes	0.178	0.363	0.001	1.249	1.596	6.616
Renal disease	0.281	0.376	0.005	1.325	0.634	2.771
DD	0.231	0.411	0.003	1.426	1.532	7.662
GLS	0.491	0.390	0.008	1.634	0.761	3.509
LA strain	1.115	0.365	0.001	2.238	1.584	6.620
HM	1.023	0. 401	0.001	1.471	1.459	4.456
Homocysteine	0.067	0.356	0.002	1.936	0.465	1.881
PON1	0.144	0.304	0.006	1.155	0.636	2.096
Constant	−1.6	0.438	0.000	0	-	-

Legend: variable(s) used in logistic regression analysis: Gender, Age, Smoker, Obesity, Diabetes, Renal disease, DD (diastolic dysfunction), GLS (global longitudinal strain), LA strain (left atrium strain), HM (Heart Model—3D ejection fraction), Homocysteine, PON1 (Human paraoxonase 1).

**Table 5:** Discriminative performance.

Model	AUC-ROC (95% CI)	Sensitivity (%)	Specificity (%)	Accuracy (%)	*p*-Value
Logistic regression	0.78 (0.72–0.83)	72	70	71	Reference
Random forest	0.86 (0.81–0.90)	80	78	79	0.01
XGBoost	0.88 (0.83–0.92)	82	80	81	0.005

**Table 6:** CV risk scores (values and standard deviation). Evaluation of different risk score in our study.

*	C		P_1_		P_2_		P_3_	
	♀	♂	♀	♂	♀	♂	♀	♂
PulsIn	11.7 ± 9.48	17.34 ± 12.3	18 ± 1.5	43 ± 9.7	26.69 ± 9.21	41 ± 9.2	34.53 ± 8.27	44.11 ± 9.68
Framingham	4.21 ± 3.4	9.09 ± 7.76	26.65 ± 6.97	33.5 ± 7.22	19.07 ± 6.75	26.18 ± 8.16	19.53 ± 2.04	25.2 ± 5.95
Qrisk 2	7.34 ± 5.92	14.1 ± 9.01	33.71 ± 8.82	40.1 ± 7.5	24.82 ± 8.63	33.2 ± 8.7	26.04 ± 3.97	32.55 ± 8.05
Score2 & Score2 OP	5.83 ± 4.7	7.76 ± 4.21	13.9 ± 7.38	15.9 ± 7.56	14.98 ± 3.47	20.3 ± 3.45	24.93 ± 3.67	22.1 ± 2.71

* Scores express in percentile as median values.

## Data Availability

The original contributions presented in this study are included in the article. Further inquiries can be directed to the corresponding author.
